# Dasatinib Associated Pleural Complications‐ A Case Series

**DOI:** 10.1002/rcr2.70532

**Published:** 2026-03-06

**Authors:** Vishnu Vazhoor, Shruthi Keechilat Pavithran, Manoj Unni, Asmita Mehta, Keechilat Pavithran

**Affiliations:** ^1^ Department of Respiratory Medicine Amrita Institute of Medical Science and Research Centre Kochi India; ^2^ Department of Pathology Amrita Institute of Medical Science and Research Centre Kochi India; ^3^ Department of Clinical Haematology Amrita Institute of Medical Science and Research Centre Kochi India; ^4^ Department of Medical Oncology Amrita Institute of Medical Science and Research Centre Kochi India

**Keywords:** adverse drug reaction, chronic myeloid leukaemia, chylothorax, Dasatinib, pleural effusion

## Abstract

Dasatinib, a second‐generation tyrosine kinase inhibitor, is highly effective in chronic myeloid leukaemia (CML) but is associated with pleural complications in approximately 28%–37% of patients. We report four patients with CML who developed dasatinib‐induced pleural effusion after prolonged treatment (2–10 years), including one case of chylothorax. All presented with dyspnea, and imaging with diagnostic thoracentesis confirmed exudative pleural effusions. One patient demonstrated triglyceride‐rich fluid consistent with chylothorax. Dose reduction of dasatinib to 50 mg/day led to complete clinical and radiological resolution in three patients while maintaining molecular remission. The fourth patient encountered recurrent chylothorax despite dosage modification and supportive therapy, necessitating permanent discontinuation of dasatinib and transition to nilotinib, resulting in sustained radiological resolution and continued molecular response. This case series highlights the spectrum of late‐onset dasatinib‐related pleural complications and demonstrates that timely dose modification or TKI switching can effectively manage toxicity while preserving oncological outcomes.

## Graphical abstract

Dasatinib is an effective therapy for chronic myeloid leukaemia but may lead to pleural complications, including exudative effusions and rare chylothorax. This case series describes four patients who developed pleural effusions after long‐term dasatinib therapy, highlighting successful management through dose reduction or TKI switching. Early recognition and tailored intervention allowed all patients to maintain or regain molecular remission while achieving radiological resolution.

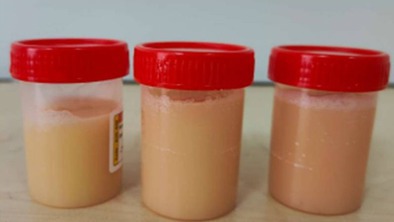

## 1 Introduction

Dasatinib is a second‐generation tyrosine kinase inhibitor (TKI) widely used in the treatment of chronic myeloid leukaemia (CML), either as first‐line therapy or in patients resistant or intolerant to imatinib [1]. Although generally well tolerated, dasatinib is associated with pleural complications, most commonly pleural effusions, with a cumulative incidence reported between 28% and 37% over the duration of therapy [2].

The pathogenesis of dasatinib‐related pleural effusion remains incompletely understood [3]. Proposed mechanisms include off‐target inhibition of SRC family kinases, platelet‐derived growth factor receptor‐β (PDGFR‐β), and immune‐mediated endothelial dysfunction [4]. Risk factors include advanced age, cardiovascular comorbidities, higher dasatinib doses, and prolonged duration of therapy [5].

Chylothorax is an exceedingly rare manifestation of dasatinib toxicity, with only a limited number of cases reported in the literature [3]. We present four cases of late‐onset dasatinib‐associated pleural disease, including one chylothorax, emphasizing diagnostic challenges, management strategies, and long‐term outcomes.

## 2 Case Series

### 2.1 Case 1

A 49‐year‐old female was diagnosed with chronic myeloid leukaemia (CML) in 2014 and was initiated on imatinib 400 mg once daily. Despite initial therapy, her BCR‐ABL ratio remained elevated (38% in September 2015), prompting a switch to dasatinib 100 mg once daily. She achieved a major molecular response (MMR), with BCR‐ABL decreasing to 0.01% by June 2019.

**FIGURE 1:**
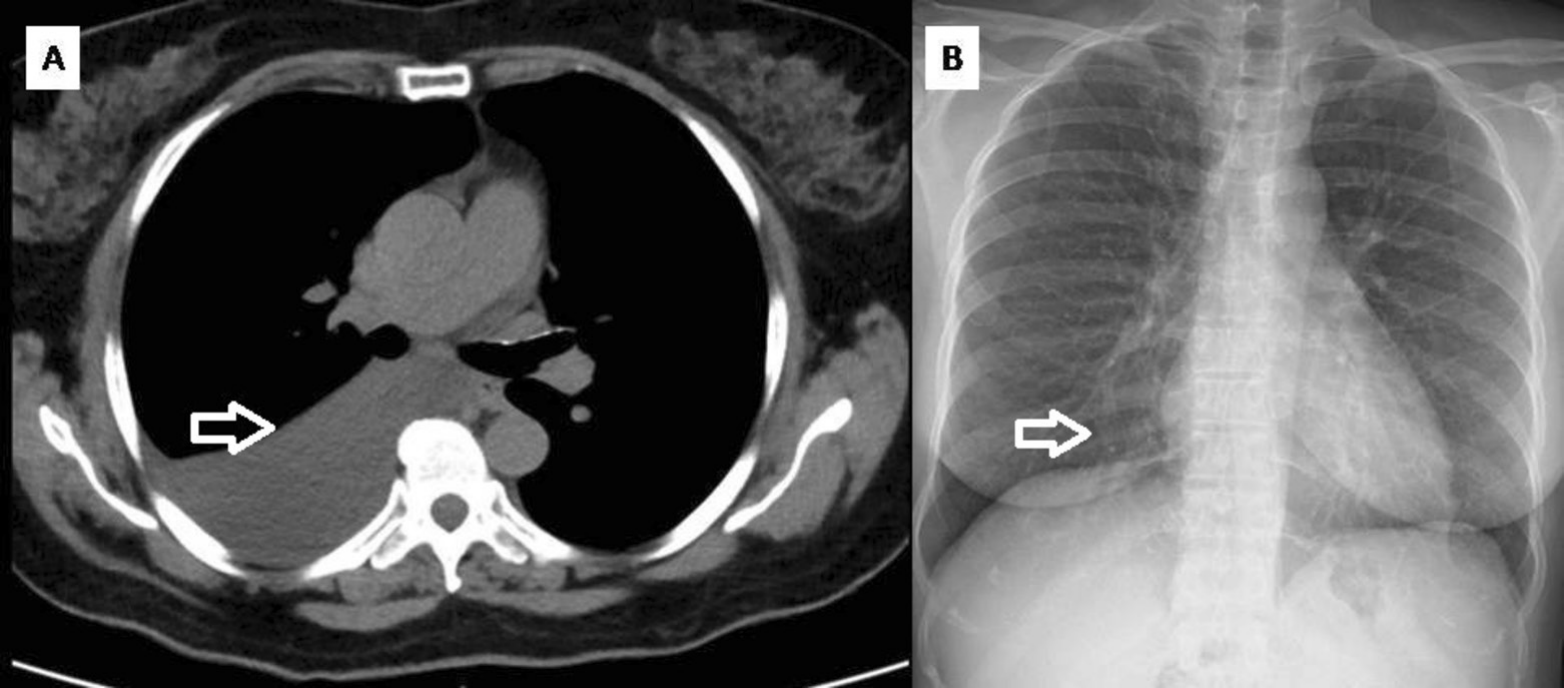
Case 1 (A) Contrast Window showing Right Pleural Effusion, (B) Latest chest X‐ray showing resolution of effusion.

In June 2024, the patient presented with a shortness of breath. On examination, bilateral pedal edema was present, while no ascites or jugular venous distension was noted. There was no significant recent weight gain. Cardiovascular examination was otherwise unremarkable. Transthoracic echocardiography demonstrated normal biventricular function with no pericardial effusion or features of pulmonary hypertension. BNP levels were not available. Chest radiography revealed right‐sided pleural effusion which was confirmed on chest CT (CT) (Figure 1A). The lung parenchyma was normal, and there were no mediastinal nodes. Diagnostic thoracentesis revealed 400 mL of straw‐coloured fluid. Laboratory analysis revealed the following: glucose, 94 mg/dL; ADA, 10.2 IU/L; LDH, 362 IU/L; Protein, 5.1 g/dL, consistent with an exudative effusion based on Light's criteria using available pleural fluid parameters, as paired serum values were not available. Cytology was negative for malignant cells, and GeneXpert for *Mycobacterium tuberculosis* was negative. Given the absence of alternative etiologies and sustained deep molecular response, the effusion was attributed to dasatinib. The dasatinib dose was reduced to 50 mg/day. A chest radiograph taken after 1 month showed a reduction in effusion, and follow‐up chest radiography in December 2024 showed the complete resolution of the effusion (Figure 1B). BCR‐ABL levels were undetectable in June 2025, and the patient tolerated the lower dose without recurrence of pleural effusion and continued to show a complete molecular response. Supportive therapy was limited to a single diagnostic thoracentesis. As the patient did not exhibit significant respiratory symptoms, additional measures such as diuretics, corticosteroids, or dietary modification were not required. Following confirmation of a drug‐related effusion, dasatinib dose reduction was undertaken and the patient was monitored with serial chest radiographs, without the need for repeat pleural intervention.

### 2.2 Case 2

**FIGURE 2:**
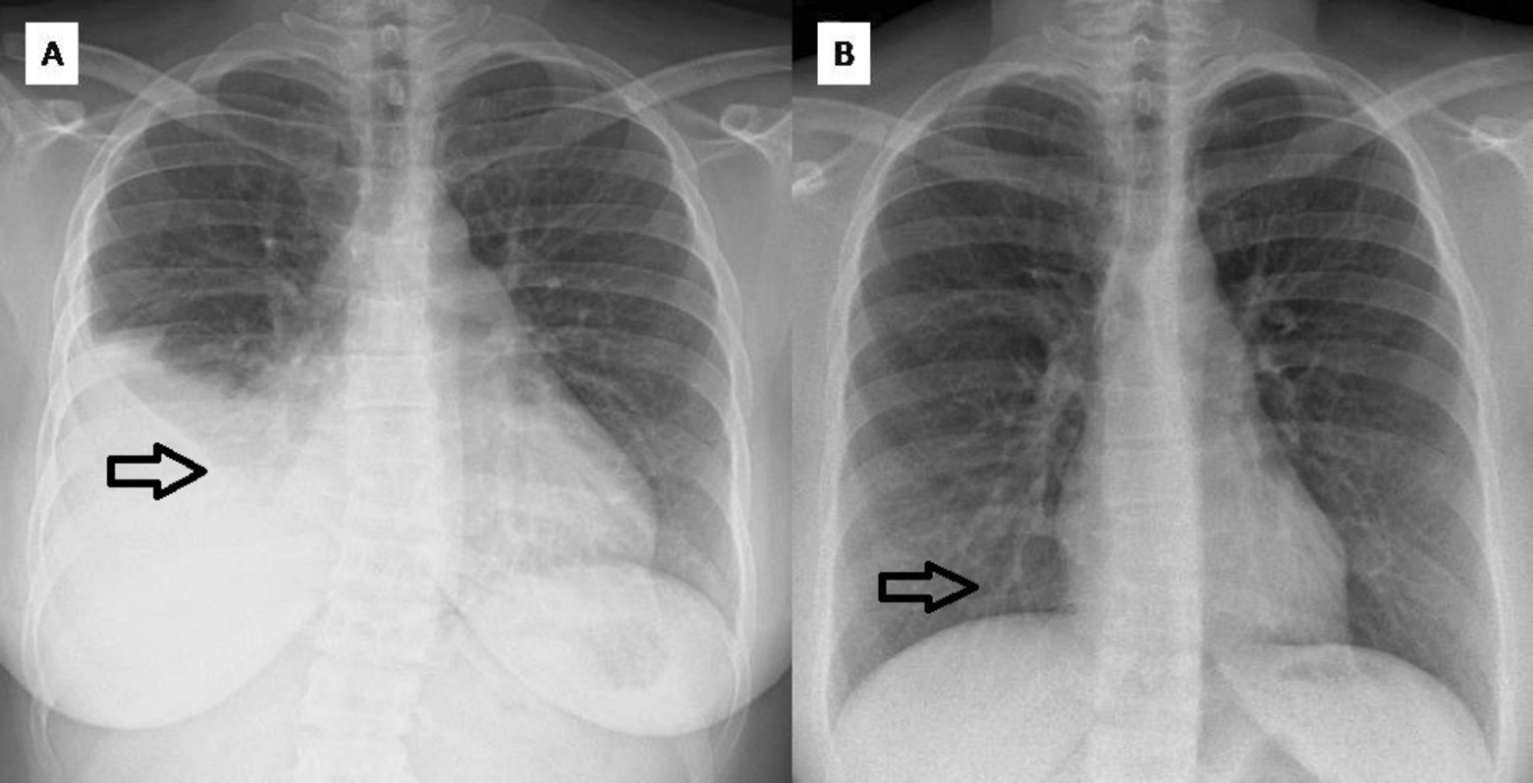
Case 2 (A) Chest X‐Ray showing right‐sided pleural effusion, (B) Follow‐up chest X‐ray with no recurrence of pleural effusion after dose reduction of dasatinib.

An 18‐year‐old female was diagnosed with CML in the chronic phase and maintained an undetectable BCR‐ABL level for nearly a decade on imatinib 400 mg once daily. After stopping treatment in May 2022 owing to sustained remission, she experienced a molecular relapse by August 2022 (BCR‐ABL: 5.28%). She was started on dasatinib (100 mg daily) in August 2022. A gradual decline in BCR‐ABL was observed, reaching 0.0003% in April 2024. In October 2024, the patient developed a right‐sided pleural effusion (Figure 2A). Diagnostic thoracocentesis drained 600 mL of straw‐coloured fluid. Pleural fluid analysis showed: total cell count: 1200 cells/μL, glucose 90.2 mg/dL; ADA 11.9 IU/L; LDH 442 IU/L; Protein 4.70 g/dL, consistent with an exudative pleural effusion. Paired serum values were unavailable for pleural fluid interpretation. Cytology was negative for malignant cells, and microbiological evaluation was unremarkable. The dasatinib dose was reduced to 50 mg daily. Given the absence of dyspnoea or hemodynamic instability, management was conservative after the initial thoracentesis. No diuretics, corticosteroids, or dietary interventions were prescribed. The patient was followed clinically and radiologically after dasatinib dose reduction, and repeat thoracentesis was not indicated. She achieved a stable disease state with no recurrence of effusion (Figure 2B). In January 2025, the BCR‐ABL was 0.003%.

### 2.3 Case 3

A 24‐year‐old male was diagnosed with chronic‐phase chronic myeloid leukaemia (CML) in July 2021 with an initial BCR‐ABL transcript level of 42.6% and was initiated on dasatinib 100 mg once daily. He demonstrated a progressive molecular response and maintained an undetectable BCR‐ABL level for nearly 2 years on therapy.

**FIGURE 3:**
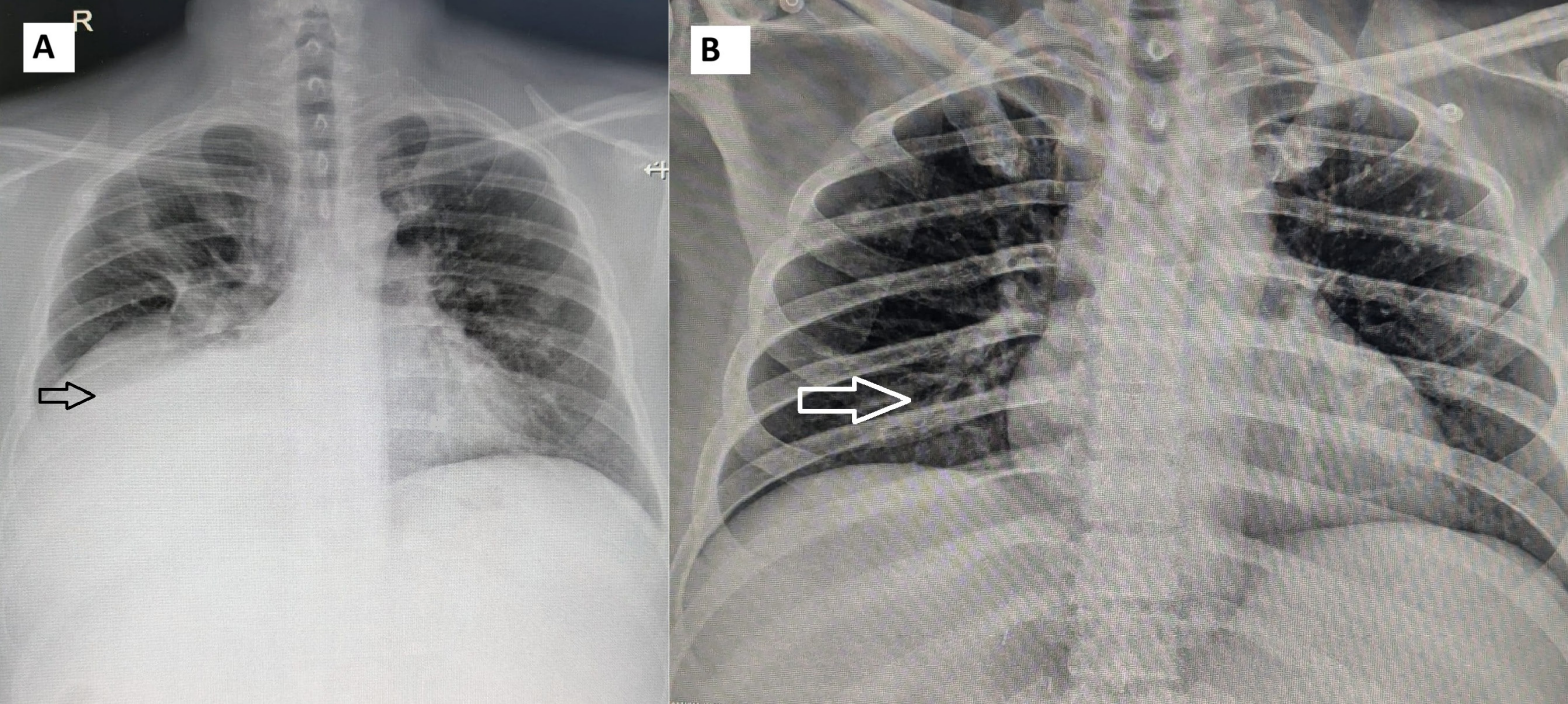
Case 3 (A) Chest X‐Ray showing right‐sided pleural effusion, (B) Follow‐up chest radiograph showing complete radiological resolution.

In June 2024, the patient developed a right‐sided pleural effusion (Figure 3A). He was clinically stable and did not report dyspnoea, chest pain, or other respiratory symptoms. Diagnostic thoracentesis was performed, draining 850 mL of straw‐coloured pleural fluid. Pleural fluid analysis revealed a total cell count of 2300 cells/μL, glucose 85.2 mg/dL, adenosine deaminase (ADA) 12.1 IU/L, lactate dehydrogenase (LDH) 395 IU/L, and protein 4.40 g/dL, consistent with an exudative pleural effusion. Serum biochemical parameters were not available for correlation. Cytological examination was negative for atypical or malignant cells, and microbiological evaluation was unremarkable.

The differential diagnosis initially considered disease progression, infective causes (including tuberculosis), and systemic causes of pleural effusion. In view of sustained deep molecular response, absence of clinical or radiological features suggestive of disease progression, low ADA levels, negative cytology, and lack of evidence of infection or systemic illness, the effusion was attributed to dasatinib‐induced pleural effusion.

No diuretics, corticosteroids, or dietary modifications were administered. Following the initial thoracentesis, the dasatinib dose was reduced to 50 mg once daily, and the patient was monitored clinically and with serial chest radiographs (Figure 3B). As the patient remained asymptomatic, repeat thoracentesis was not required.

In June 2025, the BCR‐ABL transcript level was 0.001%, confirming sustained molecular remission without disease progression, with no clinical or radiographic evidence of recurrent pleural effusion.

### 2.4 Case 4

A 51‐year‐old female with Chronic Myeloid Leukaemia, diagnosed in 2014, was initially treated with imatinib but transitioned to dasatinib 100 mg daily in 2016 owing to poor response. She achieved sustained molecular remission (BCR‐ABL reduced to < 0.01%) by 2019. However, the patient was lost to follow‐up until 2023.

**FIGURE 4:**
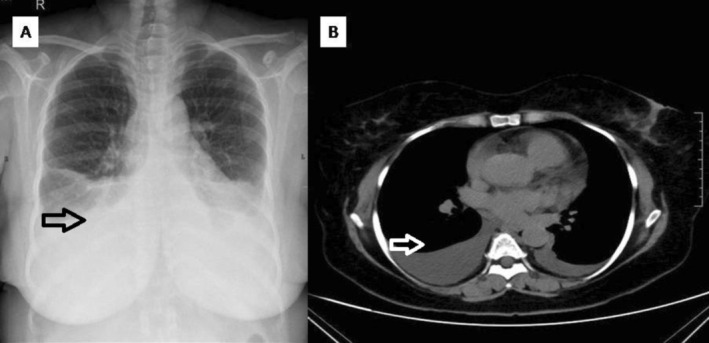
Case 4 (A) Chest radiograph showing bilateral costophrenic angle blunting. (B) Contrast‐enhanced CT showing right moderate and left pleural effusion.

She presented with 2 months of dyspnea (mMRC grade 1), accompanied by back and abdominal discomfort. Chest radiography showed bilateral CP angle blunting (Figure 4A). Ultrasound of the chest revealed mild left pleural effusion and moderate right pleural effusion, with contrast‐enhanced computed tomography confirming the same with no evidence of lymphadenopathy (Figure 4B).

**FIGURE 5:**
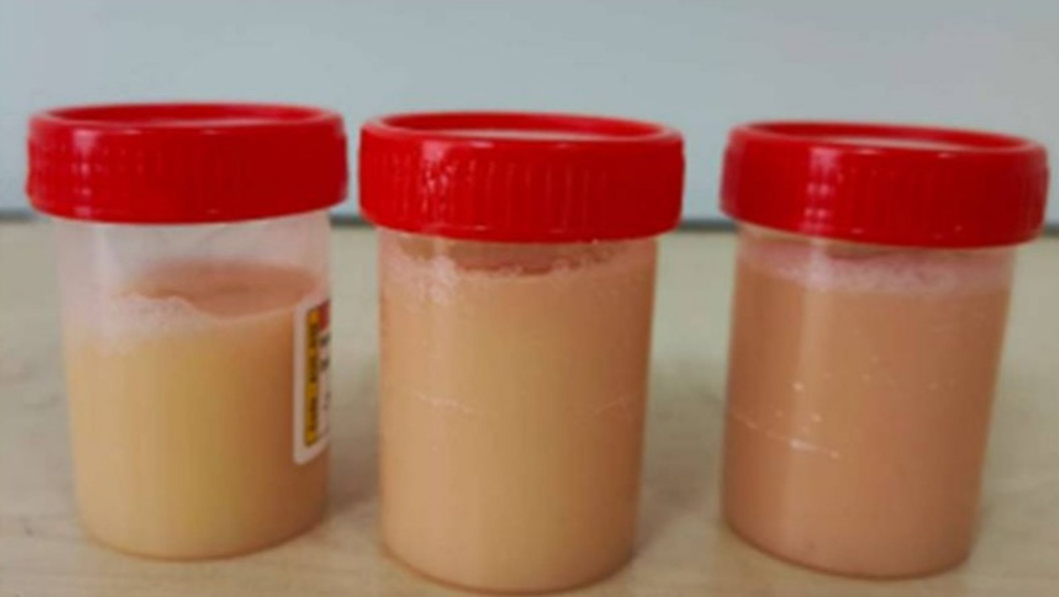
Case 4. Pleural fluid aspiration showing milky‐white fluid.

Pleural fluid aspiration was performed (Figure 5). Analysis showed: pH 7.5, total cell count: 3500 cells/μL, glucose‐119 mg/dL, protein‐4.3 g/dL, albumin‐3.04 g/dL, triglycerides‐567.5, cholesterol‐52, confirming chylothorax. Cytology: Lymphocyte rich, and negative for atypical cells. GeneXpert/AFB smears and AFB cultures for *Mycobacterium tuberculosis* were negative.

**FIGURE 6:**
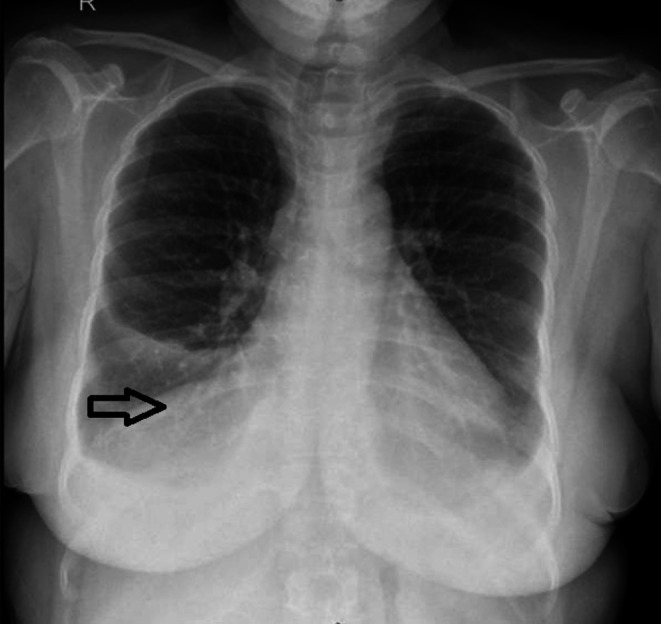
Case 4. Follow‐up chest X‐ray confirming radiological resolution.

Lymphoscintigraphy did not demonstrate a clear site of chyle leak. Dasatinib was withheld for 2 weeks. Supportive management included a low‐fat medium‐chain triglyceride diet and diuretics. In addition, oral prednisolone at a dose of 0.5 mg/kg/day was administered for 2 weeks, followed by tapering over the subsequent 2 weeks, given the suspicion of drug‐induced inflammatory endothelial dysfunction contributing to chylothorax, despite steroids not being standard therapy for non‐surgical chylothorax. BCR‐ABL on May 2023 was 0.0058%. Dasatinib was reintroduced at 50 mg/day, later increased to 70 mg/day. However, recurrent pleural effusion developed, necessitating the discontinuation of dasatinib. She was switched to nilotinib 400 mg twice daily in September 2023, with complete radiological resolution (Figure 6) and sustained molecular remission (BCR‐ABL undetectable in June 2025).

## 3 Discussion

Dasatinib, a second‐generation tyrosine kinase inhibitor (TKI), has significantly improved outcomes in chronic myeloid leukaemia (CML), particularly in imatinib‐resistant or intolerant patients. However, its use is associated with pleural complications, particularly pleural effusions and chylothorax [6, 7]. The mechanism underlying dasatinib‐induced pleural effusion remains unknown. The proposed mechanisms include off‐target inhibition of SRC family kinases, platelet‐derived growth factor receptor beta (PDGFRβ), and immune‐mediated endothelial dysfunction [4]. Pleural effusion rates were greater in leukaemia patients who underwent dasatinib therapy and developed lymphocytosis [8].

The development of chylothorax, as seen in Case 4, is an extremely unusual presentation. One possible pathogenesis theory is that dasatinib inhibits several groups of tyrosine kinases. PDGFR‐β regulates angiogenesis, lymphangiogenesis, capillary pericyte recruitment, and mesangial and vascular smooth muscle cell proliferation. Its suppression impairs vascular remodelling and causes microscopic abnormalities in lymphatic channels, resulting in chylous effusions [3]. Dasatinibis the TKI most frequently associated with chylothorax; rare cases with mTOR inhibitors (sirolimus and everolimus) have also been reported [9, 10].

Clinical manifestations range from asymptomatic radiological findings to severe dyspnoea and large effusions that require intervention. In our series, all cases were exudative, and one developed chylothorax. Chylothorax is particularly uncommon and has been reported in only 22 cases [3, 9, 11]. Management strategies include temporary discontinuation of dasatinib, dose reduction, diuretics, corticosteroids, and thoracocentesis. In refractory or recurrent cases, switching to an alternative TKI, such as nilotinib or bosutinib, is recommended [12]. Dasatinib cessation due to associated pleural effusions was required only in a small percentage of patients (6%–7%) with chronic phase CML with proper clinical care (including dose reductions, discontinuation, and pharmacologic treatments) [6] In our patients, dose reduction led to symptom resolution in three cases, while the fourth required permanent discontinuation and a switch to nilotinib due to recurrent chylothorax. In most cases, the molecular response was maintained or regained even after dose reduction or TKI switching, suggesting that treatment efficacy can be preserved with appropriate management [13]. Close monitoring of pleural status through imaging and clinical follow‐up is essential, especially after 6–12 months of therapy when the risk increases [14]. Predictors of pleural effusion included advanced age, history of cardiac or pulmonary comorbidities, and initial and higher dasatinib doses [14]. Notably, three patients in our series were female and were on prolonged dasatinib therapy, highlighting the importance of dasatinib duration as a risk factor (Table 1). Dasatinib‐induced chylothorax in adults has not been treated with fasting, in contrast to the standard care of chylothorax [15]. Ascites was absent in dasatinib‐related pleural effusion cases; however, there was a low‐grade pericardial effusion. This behaviour may be explained by the simultaneous activation and inhibition of kinases, among other theories [15]. Patients with and without pleural effusion showed comparable overall responses to dasatinib, progression‐free survival, and overall survival [2].

**TABLE 1:** Summary comparison of clinical features, diagnostic findings, management, and outcomes of the four cases of dasatinib‐associated pleural complications.

Feature	Case 1	Case 2	Case 3	Case 4
Age/Sex	49/F	18/F	24/M	51/F
Duration of dasatinib before effusion	9 years	2 year	3 years	7 years
Effusion type	Exudative	Exudative	Exudative	Chylothorax
Laterality	Right	Right	Right	Bilateral
Management	Dose reduction	Dose reduction	Dose reduction	withhold → stop + steroids + diet + diuretics
Initial TKI	Imatinib 400 mg od	Imatinib 400 mg od	Dasatinib 100 mg od	Imatinib 400 mg od
Outcome	Resolved	Resolved	Resolved	Resolved after TKI switch
Current TKI	Dasatinib 50 mg once daily	Dasatinib 50 mg once daily	Dasatinib 50 mg once daily	Nilotinib 400 mg twice daily
Recurrence	No	No	No	Yes
Initial BCR‐ABL level	38%	5.28%	42.6%	< 0.01%
Latest BCR‐ABL	Undetectable	0.003%	0.001%	Undetectable

In conclusion, pleural effusions are a well‐recognised complication of long‐term dasatinib therapy and can arise even after prolonged exposure, consistent with prior reports of pleural fluid accumulation in this setting. Early identification and tailored management, including judicious dose reduction or switching to alternative TKIs when required, allow effective control of pleural disease without compromising durable molecular remission. Our case series highlights that a conservative approach, guided by clinical severity and systematic evaluation of fluid retention, can be successful in selected patients. Clinicians should maintain vigilance for this adverse effect throughout the course of dasatinib therapy, as timely intervention promotes optimal outcomes while preserving therapeutic efficacy.

## Author Contributions

Conception and design: all authors. Collection and assembly of data: all authors. Manuscript writing: Vishnu V, Shruthi K.P., Final approval of manuscript: all authors.

## Funding

The authors have nothing to report.

## Ethics Statement

This case series was conducted in accordance with the ethical standards of the institutional review board and with the principles of the Declaration of Helsinki.

## Consent

The authors declare that written informed consent was obtained from all the patients for the publication of this manuscript and accompanying images using the consent form provided by the Journal.

## Conflicts of Interest

All authors declare no conflicts of interest.

## Data Availability

The authors have nothing to report.
